# Therapeutic Potential of Cannabis: A Comprehensive Review of Current and Future Applications

**DOI:** 10.3390/biomedicines11102630

**Published:** 2023-09-25

**Authors:** Zach J. Leinen, Rahul Mohan, Lakmini S. Premadasa, Arpan Acharya, Mahesh Mohan, Siddappa N. Byrareddy

**Affiliations:** 1 Department of Pharmacology & Experimental Neuroscience, University of Nebraska Medical Center, Omaha, NE 68182, USA; zleinen@unmc.edu (Z.J.L.); ramohan@unmc.edu (R.M.); arpan.acharya@unmc.edu (A.A.); 2 Southwest National Primate Research Center, Texas Biomedical Research Institute, San Antonio, TX 78227, USA; lpremadasa@txbiomed.org (L.S.P.); mmohan@txbiomed.org (M.M.)

**Keywords:** cannabis, cannabinoids, gut-brain axis, inflammation, THC, CBD, SARS-CoV-2, monkeypox

## Abstract

Historically, cannabis has been valued for its pain-relieving, anti-inflammatory, and calming properties. Ancient civilizations like the Egyptians, Greeks, and Chinese medicines recognized their therapeutic potential. The discovery of the endocannabinoid system, which interacts with cannabis phytoconstituents, has scientifically explained how cannabis affects the human immune system, including the central nervous system (CNS). This review explores the evolving world of cannabis-based treatments, spotlighting its diverse applications. By researching current research and clinical studies, we probe into how cannabinoids like Δ9-tetrahydrocannabinol (THC) and cannabidiol (CBD) help to manage conditions ranging from chronic pain, persistent inflammation, cancer, inflammatory bowel disease, and neurological disorders to even viral diseases such as Human Immunodeficiency virus (HIV), SARS-CoV-2. and the emerging monkeypox. The long-term recreational use of cannabis can develop into cannabis use disorder (CUD), and therefore, understanding the factors contributing to the development and maintenance of cannabis addiction, including genetic predisposition, neurobiological mechanisms, and environmental influences, will be timely. Shedding light on the adverse impacts of CUD underscores the importance of early intervention, effective treatment approaches, and public health initiatives to address this complex issue in an evolving landscape of cannabis policies and perceptions.

## 1. Introduction

**Figure 1:**
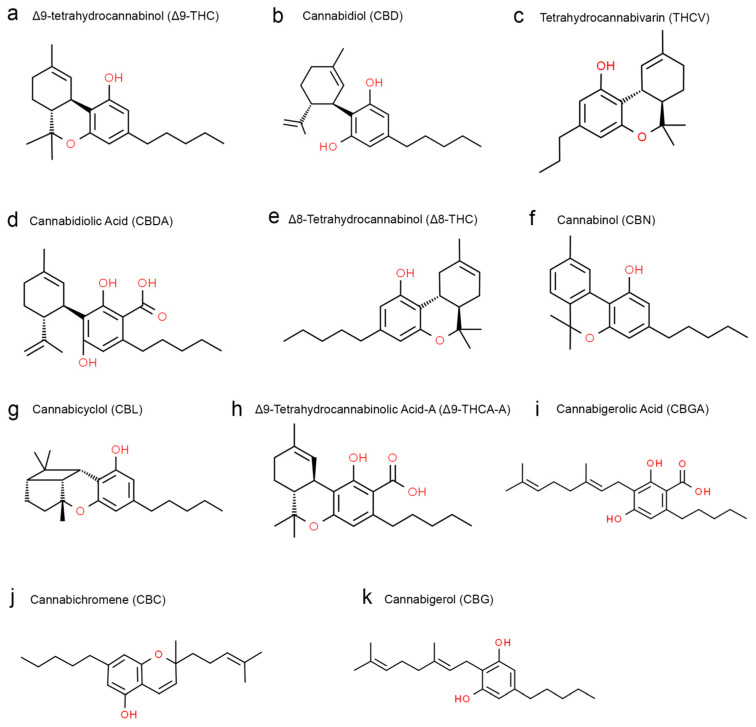
Structural formulas of different phytocannabinoid types from the cannabis plant obtained from the ChemSpider|Search and share chemistry (chemspider.com (accessed on 10 August 2023)).

*Cannabis sativa*, commonly known as cannabis, has been used for years for recreational and, more importantly, therapeutic or medicinal purposes [1]. The cannabis plant contains a variety of phytochemicals, including flavonoids, terpenoids, phytocannabinoids, alkaloids, glycoproteins, and phytosteroids [2] (Figure 1). The most well-known and studied phytochemicals in cannabis are the cannabinoids. Δ9-tetrahydrocannabinol (THC) is the primary psychoactive cannabinoid, produced mainly in the leaves and flower buds of the plant [2]. Besides THC, there are also non-psychoactive cannabinoids with several medicinal properties, such as cannabidiol (CBD), cannabichromene (CBC), cannabigerol (CBG), etc. along with other non-cannabinoid constituents [3]. Different parts of the cannabis plant have different concentrations of these phytochemicals. For example, leaves and glandular trichomes in the bark contain the most potent cannabinoid metabolites. Flower buds are primarily rich in THC, CBD, CBC, and CBG [4]. Two clinically relevant cannabinoids that have been well studied include Δ9-tetrahydrocannabinol (THC), the most abundant cannabinoid that is anti-inflammatory but has psychoactive properties, and cannabidiol (CBD), which exerts anti-inflammatory and anxiolytic effects and is non-psychoactive [2]. Endogenously produced cannabinoids, endocannabinoid receptors, and their metabolic enzymes that together comprise the endocannabinoid system (ECS) regulate many processes controlled by the central nervous system, such as mood, hunger, memory, pain and neurogenesis [5]. The cannabinoids interact with receptors such as cannabinoid receptor-1 (CB1) and cannabinoid receptor-2 (CB2) to signal through the ECS to trigger a response. Specifically, endocannabinoids activate CB1 and CB2 to regulate the release of neurotransmitters like acetylcholine, dopamine, gamma-aminobutyric acid (GABA), glutamate, serotonin, norepinephrine, and endogenous opioids [6,7].

**Figure 2:**
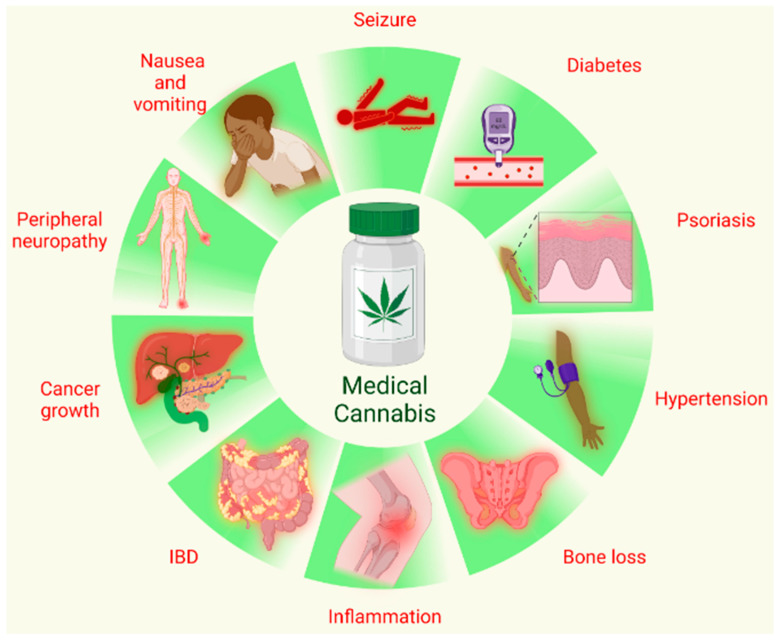
Medical use of cannabis. Cannabis and different phytocomponents of cannabis, like THC and CBD, have been used for various therapies that include but are not limited to treating seizures and convulsions, vomiting and nausea, peripheral neuropathy and pain, and psoriasis; reducing cancer cell growth, bone loss, and inflammatory bowel disorder; being used as an anti-inflammatory and anti-hypertensive; and lowering blood glucose level (Generated using BioRender).

The two known endocannabinoids include 2-arachidonoyl glycerol (2-AG) and arachidonoyl ethanolamide (AEA), also known as anandamide, which can activate G-protein coupled receptors (GPCRs), nuclear receptors, like PPARγ, GPR55 and several ion channels like TRVP1 and 4 [5]. The increasing legalization of cannabis for medicinal purposes underscores the need for further research to understand both the beneficial and harmful effects of cannabis and cannabinoids on the immune system. Cannabinoids have shown great promise in managing chronic pain, nausea and vomiting, seizures and convulsions, peripheral neuropathy, psoriasis, reducing cancer cell growth, inflammatory bowel disorders, bone loss, hypertension, and lowering blood glucose levels. Nevertheless, further research is necessary to establish safety, efficacy, appropriate dosages, and potential interactions with other medications. The therapeutic effects/potential of cannabis/cannabinoids are summarized in Figure 2. Distinct from these, the long-term recreational use of cannabis, especially during adolescence, leads to addiction, impaired cognitive function, memory deficits, and the development of hallucinations and psychosis.

### Methodology of Literature Search

For this comprehensive review of the therapeutic potential of cannabinoids and current and future applications, a rigorous and methodical approach was taken in selecting and assessing the literature. An in-depth comprehensive literature search was conducted using multiple databases, including PubMed, Google Scholar, Research Gate, etc., to ensure the pertinence of the gathered information, The search strategy comprised relevant keywords and phrases such as cannabis for pain relief, effects of cannabis on inflammation, cannabis and cancer, cannabis and gastrointestinal diseases, the effect of cannabinoids on neurocognitive diseases, cannabinoids and HIV, gut–brain axis, cannabis addiction, cannabis and emerging viral diseases, synthetic cannabinoids, as well as the future therapeutic potential of cannabinoids. The search was performed without restrictions on publication dates, giving the highest significance for the most recent literature, including review articles, to highlight the translational impact of the included information. All the retrieved articles were thoroughly examined by the authors to determine the reliability of the data reported in the articles and their suitability for the review topic, and the authors independently extracted the information. Specific exclusion criteria were used to ensure the quality of the review, by filtering out the non-peer-reviewed materials such as blog articles, conference abstracts, and unpublished manuscripts. The authors worked collaboratively to synthesize a cohesive and informative literature analysis and present a comprehensive review of the therapeutic effects of cannabis and their current and future prospects.

While THC is psychotropic, CBD, another phytocannabinoid present in the *Cannabis sativa* plant, is non-psychotropic and has witnessed widespread acceptance for the symptomatic treatment of various medical conditions. CBD has antipsychotic, anxiolytic, anti-seizure, and anti-inflammatory properties and potentially mitigates some of the adverse psychotropic effects of THC by acting as a negative allosteric modulator of CB1 receptor [8]. Besides its beneficial effects, when taken in controlled quantities under medical supervision, cannabis overuse on the other hand has been shown to increase the risk of psychotic symptoms later in life when used chronically and in high doses [7] during adolescence. Chronic high amounts of THC lowers activity in some parts of the brain that control planning and performing cognitive tasks, while CBD has been shown to increase activity in these parts of the brain [7]. Therefore, possibility of using CBD to suppress some of THC’s adverse effects could be a viable option that requires further research. A perfect ratio of THC:CBD for therapeutic use has not been found, but identifying an optimum ratio between THC and CBD may provide a way to lower adverse effects. Accordingly, this review will discuss the clinical benefits of phytocannabinoids, their potential as therapeutics against various conditions including emerging and reemerging viral outbreaks, and the adverse impact of recreational cannabis overuse.

## 2. Cannabis and Pain Relief

Medicinal cannabis, which involves using the whole cannabis plant or its extracts, holds great promise for the clinical management of chronic neuropathic pain. Yet, concerns remain about standardization, dosing precision, potential side effects, and long-term safety [9]. At present, opioids stand as the sole medication capable of addressing chronic pain, although a range of adverse effects accompanies their usage. However, there is potential to circumvent these drawbacks by considering low-dose cannabinoids as an alternative option. Abundant CB1 receptors expressed within the brain’s hippocampus modulate internal brain signals, and it is worth noting that these receptors share structural similarities with opioid receptors. Furthermore, CB2 receptors are prominently located in the sensory neurons of the dorsal root ganglion and the spinal cord. These regions are recognized for their involvement in nociceptive integration, implying that the ECS may play a significant role in pain regulation. Cannabinoids, by targeting these receptors within the ECS, hold promise in offering an alternative pathway for managing chronic pain. The abundant CB1 receptors in the hippocampus suggest a potential for cannabis/cannabinoids to modulate cognitive and emotional aspects of pain perception, distinct from the more direct analgesic effects of opioids. Similarly, the presence of CB2 receptors in pain-associated neural pathways implies that cannabinoids could influence pain processing and perception at a fundamental level. Exploring cannabinoids as a substitute for opioids in pain management requires a thorough understanding of the complex interactions between cannabinoids and the ECS and their specific impacts on pain pathways.

A recent study by Andreae et al. using the Bayesian analysis from five randomized trials showed that inhaled cannabis provided short-term relief from chronic neuropathic pain for 15–20% of patients [10]. Dykukha et al. [11] reported that when nabiximol oromucosal spray was administered in people with non-cancer neuropathic pain, it was correlated with a statistically significant improvement in pain relief compared to a placebo group. In contrast, other groups found it not very effective for neuropathic pain. However, studies by Mücke et al. concluded that the adverse effects of cannabis-based medicines for treating neuropathic pain outweighed its potential benefits [12]. Further, Petzke et al. suggested that cannabis-based medications should be considered a third-line therapy for managing chronic pain after the first and second-line therapies have failed [13]. Another study determined the long-term effects of dronabinol on multiple sclerosis (MS) patients experiencing neuropathic pain over 32 weeks, and it was found that on a scale of 0–10, most patients said they experienced a pain level of 2.8–3.2 during the trial [14]. Another 38-week open-level trial evaluating the effects of a THC/CBD spray (oromucosal) on patients with diabetes-induced neuropathic pain found it to be well-tolerated and effective for pain management [15]. Overall, these studies exhibit mixed evidence of the usefulness of cannabis/cannabinoids in neuropathic pain management. A separate comprehensive study involving 338 patients with various chronic pain conditions investigated cannabis flos decoction as a supplementary treatment over 12 months. The results revealed statistically significant improvements in pain intensity, pain disability, anxiety, and depression compared to their status at the beginning of the study, demonstrating the effectiveness of cannabis/cannabinoids as an adjunct to existing analgesic therapies. An observational study involving 428 individuals with osteoarthritis, autoimmune arthritis, or rheumatoid arthritis assessed CBD’s efficacy for pain and arthritis symptoms. Participants were asked to rate their pain on a 0–10 scale before and after CBD consumption. Additionally, they were asked to report any changes in the doses of their other medications after CBD use. The findings showed that many participants experienced enhanced pain relief from CBD usage. The 10-point scale assessment revealed that 44% of the overall group experienced an average pain reduction of 2.58. Moreover, among respondents using CBD for joint pain, a notable 60.5% reported either reducing or discontinuing other medications due to the positive effects of CBD. Although the potential benefits are intriguing, further research is necessary to elucidate the precise mechanisms and optimize the potential of cannabis-based interventions to alleviate chronic pain effectively, while at the same time reducing possible adverse effects.

## 3. Cannabis and Inflammation

Chronic and unresolved inflammation plays a central role in the onset of many chronic diseases, such as diabetes, asthma, atherosclerosis, chronic obstructive pulmonary disease (COPD), inflammatory bowel disease (IBD), neurogenerative disease, multiple sclerosis, and rheumatoid arthritis [16]. The inflammatory response begins with innate immune cells such as neutrophils and macrophages releasing inflammatory mediators that initiate several signaling pathways [16]. Three steps occur in an acute inflammatory response: increased blood flow to the site of inflammation, vasodilation of the blood vessels in the affected area, and phagocytic leukocyte migration to the affected area and into the affected tissue [17]. The first change noticed is the decrease in vascular flow and increased size of blood vessels [17]. Alterations in the endothelium boost the permeability of the microvasculature, and the decreased fluid in the lumen allows the blood to become more viscous [17]. This makes it easy for leukocytes to adhere to the endothelium and move through the wall and exit the blood vessels [17]. If the inflammation is not resolved, it becomes chronic, which can drive various diseases and comorbid disorders [17].

In this context, CBD has been shown to reduce inflammation by stimulating the release of anti-inflammatory cytokines [16,18], thereby diminishing pro-inflammatory cytokine levels, restraining T-cell proliferation, and triggering T-cell apoptosis [19]. THC also has anti-inflammatory properties, and one study showed that the topical treatment of THC in mice with dinitrofluorobenzene (DNFB)-mediated allergic contact dermatitis effectively reduced immune cell infiltration and decreased allergic ear swelling [20]. More importantly, CBD and other similar cannabinoids have proven viable treatment options for many inflammatory diseases of the intestines, brain, and skin [21]. B cells, NK cells, neutrophils, CD8+ T cells, monocytes, and CD4+ T cells express CB1 and CB2 receptors. CB2 receptors, in particular, are abundantly expressed in human B cells, NK cells, monocytes, neutrophils, and T cells [21]. CB2 receptor expression levels on macrophages increase significantly during cell activation and inflammation, suggesting a regulatory role for the endocannabinoid system in inflammation. Accordingly, cannabinoids can be expected to exert the most significant effect during increased cellular activation and inflammation [21].

Another essential property of cannabinoids is their ability to modulate intestinal permeability and help maintain barrier integrity. In this regard, as chronic inflammation increases intestinal permeability, THC and CBD can potentially restore barrier integrity to their healthy state and, in doing so, prevent the translocation of harmful microbes and their by-products from the intestinal lumen into the systemic circulation [21,22]. Therefore, cannabis-based medicines could potentially be used to treat intestinal diseases with abnormal gut permeability and inflammation [21]. Cannabis also has a role in maintaining the diversity and relative abundance of gut microbiota. Chronic inflammation associated with gut dysbiosis results in an imbalance of *Firmicutes*: *Bacteroidetes* ratio that can trigger the onset of obesity and other metabolic syndromes. THC can reverse this by restoring the normal microbiome and blunting the development of obesity [21]. From a mechanistic standpoint, CBD has been shown to increase adenosine signaling via A2A receptors, leading to reduced cellular activation [23]. These anti-inflammatory effects of CBD were confirmed using an antagonist of the A2A receptor that prevented CBD’s ability to dampen cytokine production [23].

## 4. Cannabis and Cancer

Cannabis and its constituents have been found to have immense therapeutic potential in cancer treatment [24]. The endocannabinoid system’s role in cancer is largely unknown and understudied. Still, dysregulation of the endocannabinoid system (consisting of the primary endocannabinoids anandamide and 2-arachidonoylglycerol, as well as the cannabinoid receptors, CB1 and CB2) has been implicated in cancer development and progression [21]. Studies utilizing mice models indicated a reduction in CB1 receptor expression within colorectal carcinoma cells, while an elevation in CB1 receptor expression was observed in hepatocarcinoma and Hodgkin’s lymphoma [21]. In addition, the expression levels of CB1 receptors have been proposed to correlate with disease severity [21]. Similarly, the overexpression of CB2 receptors has been reported in human breast adenocarcinomas and gliomas.

Increasing published evidence pointing to the ability of CBD to modulate various signal transduction pathways that regulate cell proliferation, differentiation, senescence, and cell death makes it a potent natural drug candidate for further testing its direct anticancer effects beyond its demonstrated efficacy as a symptomatic medication to alleviate nausea and vomiting associated with cancer chemotherapy. [25]. Specifically, CBD has been shown to induce cell-cycle arrest from the G0 to G1 phase, directly leading to a reduction in CDK2/cyclin E protein levels [25]. This likely represents several mechanisms by which cannabinoids can modulate the cell cycle. In leukemic cells, cannabinoids use ceramide to induce apoptosis by modulating the p38 mitogen-activated protein kinase [26]. In glioma cells, cannabinoids upregulate endoplasmic reticulum stress-related genes, which induces apoptosis. Lung cancer cells undergo apoptosis when CBD upregulates the expression of cyclooxygenase-2 and prostaglandin E2 [26]. All these examples point to some of the critical mechanisms by which CBD induces cancer cell death and the potential therapeutic role of CBD as an anticancer agent. Cannabinoids also downregulate vascular endothelial growth factors to decrease proliferation [25]. Likewise, cannabinoids can also deter metastasis by inhibiting cell adhesion and migration by modifying matrix metalloproteinase 2 (*MMP2*), *MMP9*, tissue inhibitor of metalloproteinases 1 (*TIMP1*), and inducing endoplasmic reticulum stress [25].

CB1 and CB2 receptors are abundantly expressed in the colonic epithelium, submucosal myenteric plexus, and smooth muscle layers [27]. CBD suppressed colorectal cancer proliferation by signaling through CB1, TRPV1, and PPARγ receptors, and interestingly, antagonists of these receptors effectively reduced CBD’s anti-proliferative properties [27]. Similarly, THC induced cancer cell apoptosis by activating the CB1 receptor and inhibiting the P13K-AKT, RAS-MAPK cascade, including the activation of BAD [27]. A dose-dependent reduction in cell survival was demonstrated when colorectal cancer cells were exposed to THC. Nevertheless, small doses caused little to no change in cancer cell survival in vitro. In a 6-month long clinical trial that included 2970 cancer patients, with over 50% of them in stage 4 disease and treated with medical cannabis for a variety of cancers (breast (20.7%), lung (13.6%), pancreatic (8.1%), and colorectal (7.9%)), the authors reported very encouraging results. Out of the 2970 patients, the study recorded 902 (24.9%) deaths, 682 (18.8%) stopping treatment, and 1211 (60.6%) responders. Intriguingly, 95.9% of the respondents reported improved health, 3.7% reported no change, and 0.3% reported a decline in their condition [28]. In an in vitro study involving a human colorectal cancer cell line, DLD-1, rimonabant (a CB1 antagonist) induced a G2-M cell cycle arrest without induction of apoptosis [29]. In human colorectal cancer cell lines Caco-2 and HCT116, CBD protected the DNA against oxidative damage and decreased cell proliferation. Though THC and CBD are the two main phytocannabinoids that have been extensively researched, some other minor cannabinoids present in the *Cannabis Sativa* plant also have shown promise in colorectal cancer treatment. For example, Cannabigerolic acid (CBGA) and tetrahydrocannabinolic acid (THCA) have demonstrated cytotoxic effects in colorectal cancer cell lines (HCT116, CCD-18Co), while producing minimal toxic effects in normal cell lines [25].

## 5. Cannabis and Gastrointestinal Diseases

Endocannabinoids have long been shown to play a significant role in modulating gastrointestinal inflammation and mucosal barrier permeability. Endocannabinoids and their downstream signaling events have also been demonstrated to modulate the composition of the gut microbiome. Despite this knowledge about the role of the endocannabinoid system in the treatment of inflammatory bowel disease (IBD) (Crohn’s disease (CD) and ulcerative colitis (UC) represent two IBD types) symptoms, opioids have been frequently prescribed for temporary relief of IBD-induced abdominal pain. Heavy opioid use to treat IBD, however, is often associated with opioid dependency and opioid overdose-related mortality [30]. Therefore, the identification and development of alternative treatments have been and continue to be a priority in IBD research. Traditional therapies include anti-inflammatory agents such as aminosalicylic acid (5-ASA), immunosuppressants, and methotrexates frequently prescribed for significant symptom relief [31]. Antitumor necrosis factor antibodies, vedolizumab, and ustekinumab are biological agents shown to be effective in treating severe cases of IBD, but these have many adverse side effects [32] that include opportunistic infections, malignancies, and injection reactions.

Numerous studies have reported that IBD patients have been and continue to self-treat with cannabis but remain oblivious to its adverse effects. Unfortunately, most IBD patients fail to inform their physicians about their cannabis use which, when consumed heavily, can elevate their risk of developing other serious complications that may eventually require surgery [33,34,35]. An observational study on patients with CD who consumed whole cannabis or cannabis products found significant improvements in their overall quality of life and disease activity index [36]. Another study conducted to determine the differences between the effects of inhaled cannabis vs. THC-free cannabis in CD patients [37] found inhaled cannabis to be superior in lowering CD severity compared to THC-free cannabis. Interestingly, C-reactive protein levels, a plasma/serum marker of inflammation, remained the same in both groups, suggesting that THC-free cannabis (CBD) provided symptomatic relief and had little to no effect on inflammation. Taken together, the available data indicate that any IBD symptomatic therapy involving cannabis is dependent on the presence of the dominant phytocannabinoid THC, known to act through the ECS via activating the CB1 and CB2 receptors [38]. Apart from phytocannabinoids, endocannabinoids such as 2-AG and AEA also bind and activate other receptors, such as GPR55, GPR119, PPARα, PPARγ, and TRPV1, that are structurally and functionally different from CB1 and CB2 receptors [39]. These receptors/ion channels and their endocannabinoid and endocannabinoid-like ligands, together referred to as the endocannabinoidome or the extended endocannabinoid system are fully functional in the majority of the organs/organ systems, including the gastrointestinal (GI) tract.

## 6. Cannabis and Neurocognitive Diseases

Cannabinoids that comprise all three known types, such as phytocannabinoids, endocannabinoids, and synthetic cannabinoids, affect the brain by predominantly activating the CB1 and CB2 [40] receptors. The CB1 receptor is one of the most common receptors in the central nervous system, and high levels of CB1 are present in the hippocampus, basal ganglia, prefrontal cortex, and cerebellum. CB2 receptor expression levels, while low in a healthy brain, drastically increase after injury or inflammation. The CB2 receptors are also mainly expressed on activated microglia, which is pivotal in phagocytosing and removing dying cells. Activation of CB2 receptors can also release cytotoxic molecules that can lead to cell death [41,42]. The activation of the CB1 receptor decreases cytokine and chemokine release and reduces inflammation and cell death. Recent reports indicate that cannabis use is frequent in older adults and among individuals suffering from chronic degenerative/inflammatory brain disorders such as amyotrophic lateral sclerosis (ALS), multiple sclerosis (MS), Alzheimer’s disease (AD), Parkinson’s disease (PD), bipolar disorder, and schizophrenia. Over the years, there has been an uptick in the use of synthetic THC-based prescription drugs like nabiximol, dronabinol, and nabilone for treating neurological disorders such as Alzheimer’s and Parkinson’s disease [43]. ALS, a rapidly progressing neurodegenerative disease affecting the motor neurons in the spinal cord and brain stem, is characterized by muscle weakness that starts in one region of the body and gradually disseminates to other parts. While the exact causative factors remain unknown, pathologic events such as oxidative stress, interference with axonal transport by neurofilaments, glutamate-induced excitotoxicity and intracellular aggregate-mediated cytotoxicity have been proposed to drive disease progression [44]. However, there is strong evidence that inflammation plays a significant role in the progression of ALS. Available evidence suggests that ALS is associated with dysregulation of the endogenous cannabinoid system, and cannabinoid receptor agonists may help slow down the progression of ALS by decreasing inflammation [41]. Studies suggest that cannabis can slow down the onset and severity of ALS symptoms such as pain, spasticity, drooling, anorexia, and inability to sleep [45]. It is also known that in ALS patients who experience respiratory difficulties, cannabis may help by increasing bronchodilation [46,47].

PD is a progressive neurodegenerative disease mostly affecting the elderly, characterized by the loss of dopaminergic neurons in the substantia nigra, leading to dysfunction of the extrapyramidal system. The consequent reduction in dopamine levels drives the major symptoms of PD, such as slowness of movement (bradykinesia), stiffness of the limbs (rigidity), and slow rhythmic tremors. In addition, PD patients also exhibit non-motor symptoms that include psychosis, anxiety, depression, and cognitive impairment [48]. Available evidence suggests the dysfunction of the endocannabinoid system in PD patients. In preclinical studies, drugs that targeted the ECS reduced motor symptoms and slowed disease progression. Intriguingly, THC administration to parkinsonian marmosets improved physical activity and hand–eye coordination [49,50]. Other studies suggest that cannabis may enhance PD-induced motor dysfunction. Studies have also shown that smoked cannabis improved motor problems such as resting tremors, rigidity, bradykinesia, and posture [48]. More importantly, CBD treatment diminished rapid eye movement, the fourth stage of sleep characterized by heightened brain activity and dream occurrences, and ameliorated sleep behavior disorder in individuals diagnosed with PD [48]. Finally, it has been shown that combining CBD and synthetic cannabinoid agonists may reduce PD-induced motor problems and pain [51].

AD is a neurodegenerative disease associated with memory impairment followed by language and behavioral problems [52]. AD is characterized by the loss of synapses and lesions, including plaques composed of a beta-amyloid (Aβ) core [53]. In the AD brain, the levels of CB2 receptors become dysregulated, while the CB1 receptor expression levels remain unaffected [54]. Animal studies have shown that compounds that elevate endocannabinoid levels decrease the toxic effects of beta-amyloid peptide, that comprises the plaque formed in the brains of AD patients [55]. Furthermore, studies with an AD mouse model showed that a THC-CBD mix decreased amyloid beta levels and reversed learning impairments [41].

Another major disease that non-psychotropic cannabinoids such as CBD may be effective as a symptomatic therapy is schizophrenia, a neuropsychiatric disease that is among the top ten leading causes of disability in the world [56]. Schizophrenia’s core symptom groups that characterize these experiences include delusions, grandiosity, paranoia, and suspiciousness. Secondary symptoms such as social exclusion, poor camaraderie, absence of motivation, impulsiveness, and emotional detachment have a blunted effect in schizophrenia patients [57]. Interestingly, a significantly higher percentage (30–50%) of people with schizophrenia use cannabis compared to the general population (10–20%). In patients with schizophrenia, cannabis may produce beneficial effects by counterbalancing the deficits in brain function, or it is possible that people with schizophrenia may have lost their control over cannabis use [58]. The dysregulation of the endocannabinoid system with a significant upregulation in the CB1 receptor expression in the dorsolateral prefrontal cortex, cingulate cortex, nucleus accumbens, and pons have been reported. However, recent reports suggest there is inadequate data on THC and CBD and its impact on schizophrenia and therefore, needs further research [56]. Overall, it is safe to conclude from the above studies that short-term cannabis use might temporarily relieve a wide range of symptoms associated with neurological disorders. Despite the beneficial effects of mild-to-moderate cannabis use, there remains a strong possibility that long-term heavy cannabis use might adversely affect mental and physiological health [41].

## 7. Cannabis, HIV, and Gut–Brain Axis

Despite viral suppression by anti-retroviral therapy (ART), people with HIV (PWH) experience intestinal dysbiosis, epithelial barrier injury, microbial translocation, and non-AIDS-associated co-morbidities. Recreational and medical cannabis use is common in the HIV-infected population primarily to alleviate nausea, sleep disorders, muscle and skeletal pain, neuropathic pain, anxiety, and depression, and in some cases, to reduce adverse effects of ART medications [41,59]. Clinical trials have established the effectiveness of cannabinoids in alleviating HIV-related neuropathic pain and nausea, although the dosing and administration methods have varied. Acute HIV infection is associated with CD4+ T cell depletion, chronic inflammation, gut epithelial barrier dysfunction and dysbiosis, and other comorbid conditions, even in people on suppressive daily ART [34]. Gut epithelial barrier dysfunction allows the translocation of inflammatory microbial products such as lipopolysaccharide (LPS) into the systemic circulation, driving chronic immune activation and disease progression. Moreover, CD4+ T cells do not return to normal levels in the gut despite effective viral suppression by ART. These alterations and persistent inflammation lead to poor HIV disease outcomes, as it increases their risk of developing comorbidities such as HIV-associated neurocognitive disorders (HAND) and HIV-associated cardiovascular disease (HACVD) [55].

Apart from their anti-inflammatory and antioxidant properties, phytocannabinoids can stimulate the production of on-demand endocannabinoids when deficient and modulate the oral and gut microbiota [59], especially in chronic inflammatory conditions. The gut microbiota supports the functions of the intestinal epithelial barrier, organized gut-associated lymphoid tissue, and other innate immune cells in the lamina propria while protecting against the invasion of pathogenic bacteria. The gut microbiota-derived metabolites such as short-chain fatty acids and indole metabolites not only nourish and nurture the epithelial cells but also regulate the functions of the mucosal immune system. such as the gut-associated lymphatic tissues (GALT) [41]. Early in HIV infection, the massive viral replication in the GALT depletes CD4+ T cells, eliciting an inflammatory response that negatively impacts the gut microbiota composition and diversity. Depleting Th17 in the GALT reduces IL-22 production, disrupting the intestinal epithelium’s tight junctions and tissue repair pathways and processes, resulting in a leaky intestinal epithelial barrier. HIV-induced dysbiosis changes the gut microbiome composition by increasing the proportion of gram-negative bacteria such as *Enterobacteriaceae*, *Prevotella*, *Prevotella copri*, and *Erysipelotrichaceae* and decreasing the proportion of *Bifidobacterium*, and *Bacteroides*. This shift to greater levels of *Prevotella* was correlated with increased levels of *CXCL10/IP-10*, a pro-inflammatory chemokine, and decreased percentages of CD4+ T cells. Cannabis’ effect on host tissues, especially gut permeability and its subsequent indirect impact on the gut microbiome, shows significant potential for strategies to combat HIV [43].

PWH also frequently suffer from comorbidities affecting the oral cavity that we collectively define here as HIV-associated oral mucosal disease/dysfunction (HAOMD) [60,61]. HAOMD is characterized by the development of chronic periodontitis (PO), Sjogren syndrome (Sjs), Oral lichen planus (OLP), and other forms of oral malignancy. Using the preclinical simian immunodeficiency virus (SIV)-infected rhesus macaque (RM) model, we investigated changes in mRNA and microRNA expression in the oral mucosa, including the salivary microbiome, to obtain deeper insights into the pathogenesis of HAOMD [62]. Chronic THC administration successfully reduced the expression of inflammation associated with *miR-21*, *miR-142-3p*, and *miR-29b* in the oral mucosa. Most strikingly, long-term low-dose THC reduced salivary dysbiosis [63]. and increased expression of the anti-endoplasmic reticulum stress protein AGR2, an epithelial barrier protecting WFDC2, and glucocorticoid-induced anti-inflammatory TSC22D3 protein in minor salivary glands and their associated ducts of SIV-infected RMs [62].

In a separate study focused on the intestine, the simultaneous profiling of miRNA and mRNA expression in the colon of the chronically SIV-infected RMs administered either vehicle (VEH/SIV) or THC (THC/SIV) identified numerous inflammation-associated miRNAs, namely, *miR-130a*,* miR-222*, and *miR-29b*, lipopolysaccharide-responsive *miR-146b-5p,* and SIV-induced *miR-190b* to be significantly upregulated in VEH/SIV but not THC/SIV RMs [22]. Compared to VEH/SIV RMs, *miR-204* was significantly downregulated in the colon of THC/SIV RMs, and in vitro studies confirmed its ability to directly target the proinflammatory extracellular matrix-degrading collagenase and neutrophil attractant *MMP8*. Moreover, THC’s ability to attenuate colonic inflammation was further evident from its capacity to inhibit the upregulation of the proinflammatory *miR-21*, *miR-141*, *miR-222*, and alpha/beta-defensins. Further, THC significantly increased the expression of tight junction proteins (occludin, claudin-3), anti-inflammatory *MUC13*, epithelial stress protecting keratin-8, epithelial proliferation associated *PROM1*, and anti-HIV chemokine *CCL5*. Furthermore, THC significantly reduced the percentages of Ki67/HLA-DR+ T cells (proliferation/activation) and PD1 (exhaustion), while increasing the percentages of CD163^+^ macrophages (M2 anti-inflammatory macrophages) in the intestine. Finally, THC significantly reduced absolute CD8^+^ T cell numbers in peripheral blood in chronic SIV infection. Most importantly, long-term low-dose THC significantly reduced collagen deposition in axillary lymph nodes’ paracortex and B-cell follicular zones, demonstrating its anti-fibrotic properties. These translational findings identified differential miRNA/gene induction and modulation of T cell activation as potential mechanisms associated with the anti-inflammatory actions of THC in the intestine that have a broader impact on not only HIV/SIV but also other chronic inflammatory diseases of the intestine [22].

Emerging evidence suggests the involvement of epigenetic mechanisms, particularly long non-coding RNAs (lncRNAs), in maintaining epithelial homeostasis. Premadasa et al. [64] recently showed that THC enhanced the interaction between long non-coding RNA *MMP25-AS1* and its associated protein coding gene *MMP25*. Enhancement of this interaction was associated with reduced neutrophil transendothelial/transepithelial migration in the colon in chronic HIV/SIV infection. Studies showed that at the transcriptional level, *MMP25-AS1* can interact directly with *MMP25*, a matrix metalloproteinase known to increase neutrophil activation and inflammation. In SIV-infected RMs treated with THC (THC/SIV), the expression of MMP25 at the protein level was further decreased by increased TIMP2 expression, an inhibitor of MMPs. This highlights THC’s potential to prevent a feed-forward mechanism for neutrophil recruitment. The reduction in *MMP25* expression was accompanied by a concurrent drop in *CXCL5* and *CCL15* expression, two potent neutrophil chemoattractants activated by *MMP25*-mediated cleavage. Importantly, THC administration to SIV-infected RMs receiving long-term ART effectively decreased *MMP25* expression in the jejunal epithelium of SIV-infected RMs, while maintaining the expression of its antisense lncRNA regulator *MMP25-AS1*. Furthermore, several other lncRNAs (*MALAT1*, *GATA6-AS1*, *GATA3-AS1*, and *SPRY-IT1*) were reported to be upregulated in response to inflammatory signaling were significantly upregulated in the colonic epithelium (CE) of VEH/SIV but not THC/SIV RMs. In contrast, fewer lncRNAs were differentially expressed in the CE of THC/SIV RMs, including *NEAT1*, *IFNG-AS1*, *MMP25-AS1*, and *BISPR*, which were significantly upregulated. Among these, *IFNG-AS1* has been shown to decrease *Salmonella enterica typhimurium* adherence and penetration by enhancing *IFNG* expression [65]. The upregulation of both *IFNG-AS1* and *IFNG* in the CE of THC/SIV RMs represents a novel epigenetic mechanism by which THC strengthens the intestinal epithelial anti-microbial defense.

In addition, we recently demonstrated that long-term low-dose THC can successfully mitigate neuroinflammation and gut dysbiosis in chronically SIV-infected RMs [66]. Specifically, chronic THC reduced the expression of type-I interferon response genes (*NLRC5*, *CCL2*, *CXCL10*, *IRF1*, *IRF7*, *STAT2*, and *BST2*) and those reported to mediate excitotoxicity (*SLC7A11* and *SLC38A2*), while concurrently enhancing protein expression of genes that reduced endoplasmic reticulum (*WFS1*) and oxidative stress (*CRYM*) in basal ganglia. Additionally, in in vitro cultured HCN2 neuronal cells, THC successfully reversed *miR-142-3p* mediated suppression of WFS1 protein expression via a CB1 receptor-dependent mechanism. Further, THC significantly elevated plasma concentrations of key endocannabinoid (AEA), endocannabinoid-like (palmitoyl ethanolamide, linoleoyl ethanolamide, and Oleoyl taurine), glycerophospholipid (glycerophosphocholine and glycerophosphoinositol) and the neuroprotective indole-3-propionate (tryptophan metabolite). Most importantly, THC reduced dysbiosis by significantly increasing the relative abundance of *Firmicutes* and *Clostridia*, including indole-3-propionate (*C. botulinum*, *C. paraputrificum*, and *C. cadaveris*) and butyrate (*C. butyricum*, *Faecalibacterium prausnitzii* and *Butyricicoccus pullicaecorum*) producers while, reducing the relative abundance of *Gammaproteobacteria* in colonic contents. Unlike other anti-inflammatory therapeutics, THC can successfully cross the blood–brain barrier and inhibit neuroinflammation due to its high lipophilicity. Overall, by reducing gut dysbiosis and simultaneously enhancing the production of endocannabinoids, endocannabinoid-like, and tryptophan metabolite levels, THC has an immense potential to be used as a therapeutic strategy to positively modulate the microbiota-gut-brain axis (MGBA) in not only HIV/SIV but also other neuroinflammatory, neurodegenerative and neuropsychiatric diseases characterized by persistent neuroinflammation and dysbiosis. In this context, a small-scale clinical trial involving ten PWH on ART confirmed the anti-inflammatory effects of oral cannabinoids (THC:CBD combo) administered for 12 weeks [67]. The findings showed promise in improving intestinal epithelial barrier function, reducing chronic immune activation, T cell exhaustion and senescence, and pathogenic events that increase the risk of developing non-AIDS-associated comorbidities. These initial, very encouraging findings are expected to pave the way for more extensive clinical trials to evaluate the therapeutic efficacy of oral cannabinoids in PWH.

## 8. Cannabis Addiction or Cannabis Use Disorder

**Figure 3:**
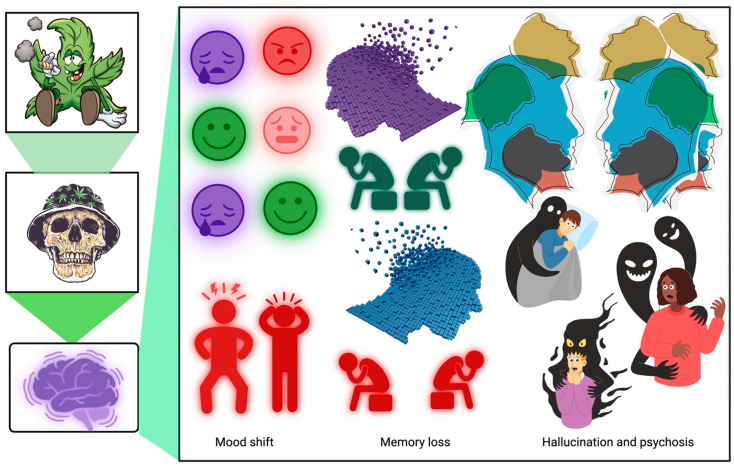
Impact of long-term recreational use of cannabis in the brain. Prolonged recreational cannabis use can lead to dependence and addiction, resulting in mood alterations, memory impairment, hallucinations, and even psychosis. (Generated using BioRender).

While cannabinoids have been widely researched and used for their anti-inflammatory and neuroprotective effects, it is essential to note that long-term exposure to cannabis smoke, THC, or CB1 receptor agonists may lead to the development of dependence, also known as cannabis use disorder (CUD). Cannabis withdrawal can also leads to bodily withdrawal signs such as abdominal constriction, wet-dog shakes, head shakes, and forepaw fluttering [68,69,70,71]. Another notable adverse effect associated with cannabis withdrawal is anxiety-like behavior that is orchestrated partly by the increased release of corticotropin-releasing hormone from the hypothalamus. Apart from these, published evidence in preclinical rodent models has shown cannabis and THC to significantly impair short- and long-term memory [72]. THC weakens the release of acetylcholine in the hippocampus and represents a likely mechanism by which it impairs memory. It was shown that memory impairment due to THC could be reversed because, after four weeks of abstinence, THC-treated rodents had returned to their baseline state. Another study showed that a single dose of THC was strong enough to impair memory three weeks after administration [52]. Other studies have shown acute cannabis use to negatively affect emotional states and significantly impair cognitive processes and gross motor functions, including memory (retrieval, working, verbal) learning, executive functions, and various attentional tasks (Figure 2). Impairments in solving math problems, time perception, and gross and fine motor skills are commonly associated with adolescent cannabis use. Further, chronic cannabis use may lead to a motivational syndrome characterized by apathy, lack of motivation, and poor educational performance (Figure 3). Also, regular cannabis use alters the structure of the gray (cell bodies, dendrites, and synapses) and white matter (myelinated neuronal tracts). Furthermore, some evidence suggests that cannabis use may increase the size of subregions of the cerebellum and amygdala in adolescents [73,74]. These changes are associated with poor executive functioning and internalizing problems. However, most studies found that cannabis use decreased the volume of brain regions [33,34,75,76,77]. In these studies, the most significant reduction in brain volume was detected in the orbitofrontal cortex, hippocampus, striatum, and amygdala. Interestingly, the degree to which the size of the hippocampus decreased depended on the amount of cannabis used and the extent of dependency [43]. Heavy cannabis use is associated with an early onset of bipolar disorder, which shows increased severity and disability. In addition, heavy cannabis use by patients with bipolar disorder has also been linked to an increased tendency to commit suicide and develop manic symptoms [41].

## 9. Cannabis and Emerging Viral Diseases

With globalization, emerging and reemerging viral outbreaks are integral to our lives. COVID-19, caused by the severe acute respiratory syndrome coronavirus-2 (SARS-CoV-2), began in late 2019 in Wuhan, China, and has since developed into a global pandemic [35]. Like other Coronavirus outbreaks, it affects the respiratory tract, leading to pneumonia-like symptoms with the potential to progress to severe acute respiratory distress syndrome (ARDS) [35,36]. The hyperinflammatory patterns of COVID-19 are like those of cytokine release syndrome (CRS). COVID-19 is driven by excessive inflammatory events, resulting in an increase in the white blood cell count but a decrease in CD4+ and CD8+ lymphocytes. This leads to an imbalance in the neutrophil-to-lymphocyte ratio [35]. After the establishment of infection, inflammatory cells enter the local site of infection, releasing pro-inflammatory cytokines, causing CRS at a very early stage of the disease. The anti-inflammatory properties of cannabis phytoconstituents could be used to prevent CRS. The endocannabinoid system controls the immune system by regulating the immune cell trafficking by acting through cannabinoid receptors [35]. Therefore, by using phytocannabinoids such as THC and CBD, attenuation of the proliferation of lymphocytes and pro-inflammatory cytokines could be a possibility [35]. Most of the severe outcomes of COVID-19 are due to CRS, and the use of medicinal cannabis could be used both as a preventative and therapeutic drug to mitigate CRS [35].

Regarding CRS, cannabinoids have been shown to down-regulate the expression of multiple pro-inflammatory genes [78]. The angiotensin-converting enzyme 2 (ACE-2) is identified as the principal entry receptor for SARS-CoV-2 in cells [79]. Once inside the lung, SARS-CoV-2 binds to the ACE-2 receptor via the spike glycoprotein and enters the cells [35]. Transmembrane serine protease 2 primes the spike proteins for SARS-CoV-2 entry into the cell by proteolysis and activation of the spike protein [35]. This leads to conformational changes and the fusion of the virus with the host membrane, allowing viral entry [35]. In in vitro studies, CBGA and CBDA were excellent allosteric and orthosteric ligands for the spike protein with affinity at micromolar levels. More importantly, both CBGA and CBDA successfully prevented the entry of SARS-CoV-2 alpha variants B.1.1.7 and B.1.351 into cells in vitro [80]. Using a combination of in vitro and in vivo approaches, Nguyen et al. [81] showed CBD and not THC to successfully inhibit SARS-CoV-2 infection of lung epithelial cells and mice. In addition to blocking viral entry, CBD also inhibited the expression of virally encoded genes and reversed the effects of SARS-CoV-2 on host gene transcription in infected cells. Follow-up studies showed that CBD exerted its anti-SARS-CoV-2 effects partly by up-regulating the *IRE1α* RNase endoplasmic reticulum stress response and interferon-stimulated signaling pathways in host cells.

Monkeypox is a contagious disease closely related to the smallpox virus in humans [82]. They both belong to the genus Orthopoxvirus [83]. The smallpox vaccine was about 85% effective against monkeypox, but after smallpox was eradicated, there was little need for continuing the smallpox vaccination programs [82]. The Democratic Republic of Congo has been the most affected country by this disease, with the initial outbreak recorded in 1970 [82]. The clinical syndrome of monkeypox is characterized by fever, rash, and lymphadenopathy, and in severe cases, it can include pneumonitis, encephalitis, light-threatening keratitis, and secondary bacterial infections [83,84,85]. Drug treatment for monkeypox is usually unnecessary, as majority of the infected individuals recover independently, but similar to SARS-CoV-2, therapeutic cannabis may help alleviate proinflammatory events and reduce disease severity [86].

## 10. Conclusions and Future Prospective

Cannabis has been used for centuries for various medicinal purposes [24]. Recently, synthetic cannabinoids have been the primary source of medicinal Cannabis, and studies have found that THC is the primary source of adverse events [24]. Unlike medical cannabis, recreational cannabis generally has a much higher THC content that can produce psychoactive effects [24]. However, cannabinoids in combination (THC: CBD) may have the potential to be an alternative to opioids for chronic pain treatment. However, research is still needed on dosing and adverse reactions that the drug combination could have on the mind and body [24]. CBD can mitigate some of the responses caused by THC [87]. Many studies have highlighted the presence of endocannabinoids (ECBs) and cannabinoid receptors (CBRs) within bone and synovial tissues, underscoring their significant roles in bone metabolism [88] and inflammation. Preclinical investigations utilizing cannabinoid-based treatments in animal models have demonstrated the potential of cannabinoids to mitigate osteoarthritis (OA) progression, prevent osteoporosis (OP), and enhance fracture healing. These findings underscore the promising therapeutic prospects of CBs in addressing various human bone-related ailments. Further, these studies also emphasize the utility of cannabinoids in treating bone loss [88]. In the majority of the cases, the medicinal use of cannabinoids is not backed by strong scientific evidence, which warrants well-designed case-controlled studies for the establishment of its beneficial role as a therapeutic [89]. Recently, the therapeutic potential of cannabinoids in alleviating neuroinflammation following traumatic brain injury (TBI) was highlighted [90]. In addition to neuroinflammation, TBI patients also develop dysbiosis [91] following neurotrauma leading to dysfunction of the MGBA [92]. Based on our recent findings [66] we believe that phytocannabinoids may be an excellent therapeutic option to restore the function of the MGBA in TBI patients and prevent the progression of neurodegeneration and cardiovascular disease risk [93].

There are over 100 cannabinoids, each with potential benefits that remain to be discovered. Some other areas of interest regarding treatment with cannabinoids include epilepsy, psychotic disorders, anxiety, and sleep disorders [8]. Data shows acute CBD administration decreases experimentally induced anxiety in healthy humans [8]. CBD is now available as an FDA-approved drug (Epidiolex^®^) for use as an anticonvulsant for refractory epilepsy (Dravet syndrome or Lennox–Gastaut syndrome), particularly in the pediatric population [94]. CBD was shown to block a positive feedback loop, wherein lysophosphotidylinositol (LPI) signaling through GPR55 produced more seizures, which resulted in increased levels of both LPI and GPR55 [95]. In vivo, mice studies demonstrated that GPR55 knockout and CBD treatment before applying seizure-inducing stimuli successfully blocked LPI-mediated effects on excitatory and inhibitory synaptic transmission. Cannabis/cannabinoids may also help treat opioid addiction. Dronabinol, a synthetic THC drug, may help treat opioid withdrawal symptoms [8]. Future research is also needed to ascertain the potential involvement and role of endocannabinoids, endocannabinoid-like terpenes, and cannabinoid receptors in mitigating systemic inflammatory responses [96].

Furthermore, there is a need for an in-depth investigation into the signaling pathways activated by cannabinoid receptors and the impact of cannabinoids on adhesion molecules, co-stimulation, and chemotaxis. This expanded research is essential for enhancing our understanding of cannabinoids and their intricate interactions with the immune system during a pathogenic insult and other immune system disorders. Collectively, emerging evidence suggests that cannabinoids may hold great promise as innovative, relatively safe, and effective anti-inflammatory agents, mainly through the targeted modulation of cannabinoid (CB2) receptors, which offers the prospect of achieving immunosuppressive effects without inducing any psychotropic adverse effects.

## Data Availability

Not applicable.

## Abbreviations

2-AG	2-Arachidonoyl Glycerol
ACE-2	Angiotensin-Converting Enzyme 2
AD	Alzheimer’s Disease
*AEA*	*Ethanolamide*
ALS	Amyotrophic Lateral Sclerosis
ARDS	Acute Respiratory Distress Syndrome
ART	Anti-Retroviral Therapy
CBD	Cannabidiol
CB1	Cannabinoid Receptor-1
CB2	Cannabinoid Receptor-2
CBGA	Cannabigerolic Acid
CBR	Cannabinoid Receptors
CD	Crohn’s Disease
CNS	Central Nervous System
COPD	Chronic Obstructive Pulmonary Disease
CRS	Cytokine Release Syndrome
CUD	Cannabis Use Disorder
ECS	Endocannabinoid System
GABA	Gamma-Aminobutyric Acid
GALT	Gut-Associated Lymphoid Tissue
GI	Gastro Intestinal
GPCRs	G-Protein Coupled Receptors.
HIV	Human Immunodeficiency Virus
HAND	HIV-Associated Neurocognitive Disorders
HACVD	HIV-Associated Cardiovascular Disease
HAOMD	Oral Mucosal Disease/Dysfunction
IBD	Inflammatory Bowel Disease
LPI	Lysophosphotidylinositol
LPS	Lipopolysaccharide
MS	Multiple Sclerosis
MMP25	Matrix Metalloproteinase 25
PD	Parkinson’s Disease
PWH	People With HIV
RM	Rhesus Macaque
SARS-CoV-2	Severe Acute Respiratory Syndrome Virus-2
SIV	Simian Immunodeficiency Virus
THC	Δ9-Tetrahydrocannabinol
THCA	Tetrahydrocannabinolic acid
UC	Ulcerative Colitis
